# P02.173. Objective and subjective adherence in mindfulness meditation trials

**DOI:** 10.1186/1472-6882-12-S1-P229

**Published:** 2012-06-12

**Authors:** H Wahbeh, B Oken

**Affiliations:** 1 Oregon Health & Science University, Portland, USA

## Purpose

Home practice is frequently prescribed as part of mind-body medicine interventions although rarely objectively measured. As part of our ongoing mind-body medicine research program, we created iMINDr, a custom monitoring software program to collect objective adherence data. The goals for these analyses were to assess: 1) any differences in objective versus subjective adherence, and 2) which baseline measures predict adherence in ongoing mindfulness meditation randomized clinical trials being conducted at our lab.

## Methods

Objective and subjective adherence data were collected from 20 participants. Measures were collected in minutes and transformed into percent out of total minutes possible. Objective adherence was collected with iMINDr, an iTouch (Apple Inc) software program designed to administer the mindfulness meditation intervention and record adherence. Age, gender, education, Perceived Stress, Positive and Negative Affect, depression inventory, Five Factor Personality, General Perceived Self Efficacy, Life Events Questionnaire, and Credibility/Expectancy were examined as potential predictors of adherence. A paired t-test was used to compare objective with subjective adherence. Objective and subjective adherence relationships were evaluated with Pearson’s correlation. Linear regression analysis was conducted for each potential predictor.

## Results

Participants reported that iMINDr was straightforward to use. Subjective adherence was greater than objective (86% ± 31% subjective, 76% ± 30% objective; t=-2.5 (df=19), p=.02). Objective adherence was correlated with subjective adherence (r=0.82, p=.0005). There were no predictors for objective adherence (all p’s > 0.05). Education predicted 47% of the variation of subjective adherence (F(1,19)=5.2, p=.04).

## Conclusion

The iMINDr is a simple objective method researchers can use to examine how home practice adherence affects outcomes in mind-body clinical trials. Objective adherence was strongly correlated with subjective adherence in study completers, although participants self-report more home practice time than actual practice time recorded from iMINDr. Higher education predicted increased adherence in meditation study participants.

